# Case report: A successful live birth after *in vitro* fertilization and embryo transfer in a patient with endometrial cancer who was treated conservatively

**DOI:** 10.3389/fonc.2024.1461216

**Published:** 2024-12-11

**Authors:** Yifan Fan, Hui Song, Xu Chen, Pei Zhang, Jingwen Si, Hui Dong

**Affiliations:** ^1^ Department of Obstetrics and Gynecology, School of Medicine, Nankai University, Tianjin, China; ^2^ Department of Obstetrics, Tianjin Central Hospital of Gynecology Obstetrics, Tianjin, China; ^3^ Tianjin Key Laboratory of Human Development and Reproductive Regulation, Tianjin, China

**Keywords:** early-stage endometrial cancer, fertility-sparing treatment, conservative treatment, *in vitro* fertilization and embryo transfer, pregnancy

## Abstract

**Objective:**

To describe a patient conceiving with *in vitro* fertilization and embryo transfer(IVF-ET) after conservative treatment of early stage endometrial cancer.

**Patient:**

A 31-year-old multiparous woman diagnosed with highly-differentiated (G1) endometrial adenocarcinoma (grade IA).

**Intervention(s):**

After four courses of conservative treatment each followed by hysteroscopic biopsy and endometrial curettage,assisted reproductive technology was performed.

**Main outcome measure(s):**

Successful pregnancy and delivery without residual endometrial cancer.

**Results(s):**

A healthy normal female infant with a birth weight of 3,690 g was born by cesarean section at 38 + 2 weeks’ gestation. No residual malignant cells was detected on a biopsy during cesarean section.

**Conclusion(s):**

Conservative fertility-sparing treatment are crucial for young patients with early stage endometrial cancer. Assisted reproductive technologies may be considered to assist such patients to conceive as soon as possible.

## 1 Introduction

Endometrial cancer is a prominent cause of mortality among gynecological malignancies worldwide. The latest data reports 420,242 new cases of endometrial cancer globally in 2022, ranking it as the 15th most prevalent type of cancer, with 97,704 new deaths, placing it at the 19th position (1). In China during the same year, the incidence rate of endometrial cancer was 11.25 per 100,000 individuals with a corresponding mortality rate of 1.96 per 100,000 individuals.Furthermore, there has been a continuous increase in the incidence rate of endometrial cancer from 2000 to 2018 in China along with a trend towards younger onset cases (2). Notably, a significant proportion of these young patients diagnosed with endometrial cancer have not given birth.

In cases of early-stage endometrial cancer confined to the uterine body, the standard surgical approach involves hysterectomy with bilateral adnexectomy and lymph node evaluation (3); however, this procedure results in loss of female fertility. On the one hand,due to the increasing age of women’s first childbirth and the progressively younger onset of endometrial cancer, some women have not completed their childbearing prior to diagnosis. On the other hand, in light of China’s three-child policy implemented since 2021, even those who have completed their first childbearing may still have a strong desire to preserve their fertility. Consequently, the fertility-sparing treatment of patients with endometrial carcinoma has become increasingly crucial.

Fertility preservation therapy necessitates comprehensive evaluation and strict screening of patients with fertility needs due to the potential risks of residual tumor lesions, disease recurrence and progression (1). Therefore, it is exclusively suitable for young patients with early-stage endometrial carcinoma without metastasis (4, 5). Typically, these patients exhibit adenocarcinoma tissue type characterized by highly differentiated tumor cells that express PR/ER positively. Consequently, the risk of metastasis is relatively low while the prognosis remains favorable. However, active conception should be pursued promptly following complete remission to minimize estrogen stimulation and avoid the heightened risk of recurrence.

Successful pregnancies have been documented in the literature following fertility preservation therapy in nulliparous females with endometrial cancer (6–8). In this particular case,we report a woman who was diagnosed with endometrial cancer two years after she had given birth to a baby. Due to her persistent desire for future fertility, we performed fertility preservation therapy following comprehensive multidisciplinary consultation and evaluation.Subsequently, she successfully achieved conception through assisted reproductive technology and delivered a healthy baby via cesarean section at full term.

## 2 Case description

**Figure 1:**
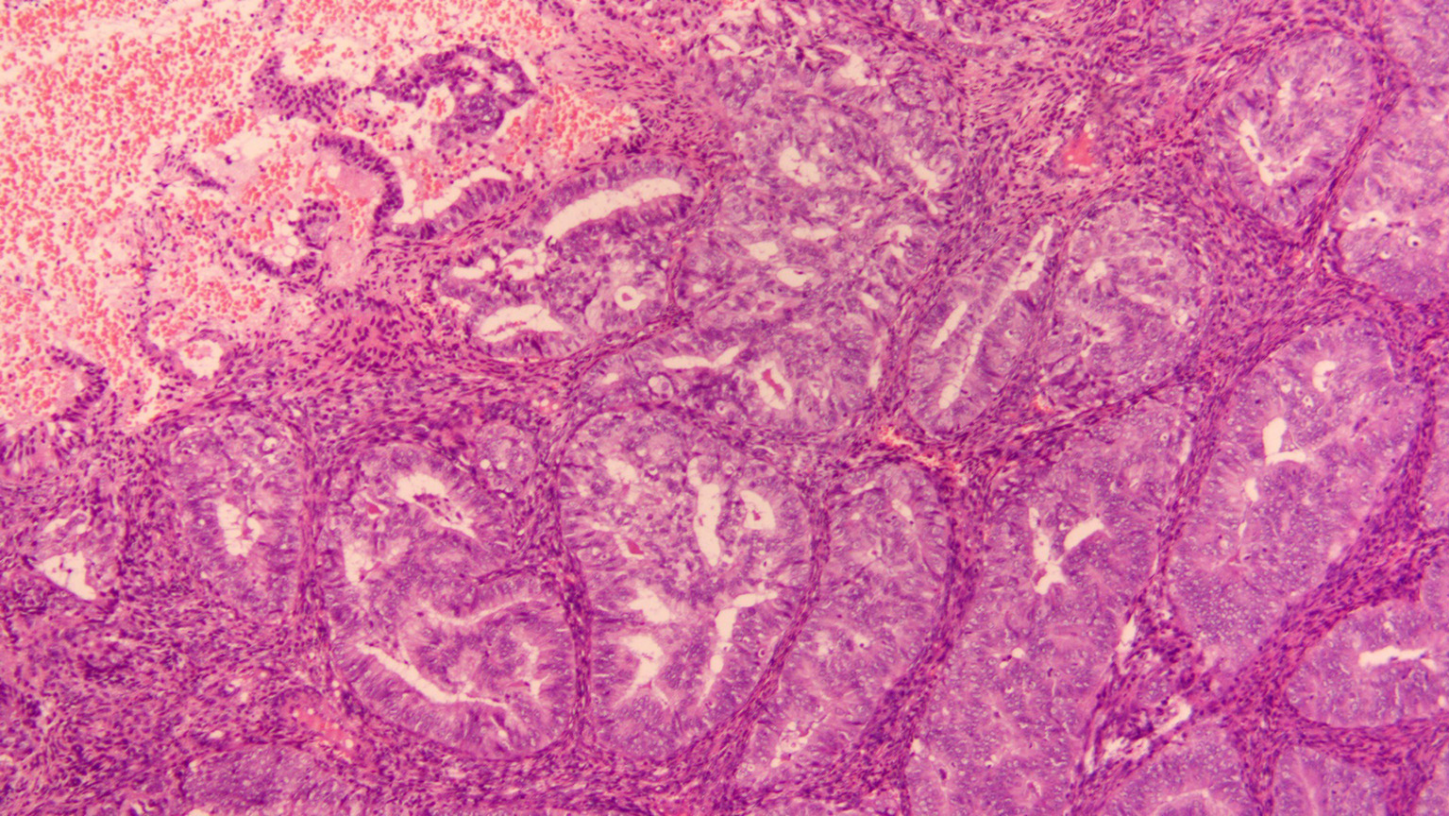
HE staining of the pathology section at diagnosis (10X).

**Figure 2:**
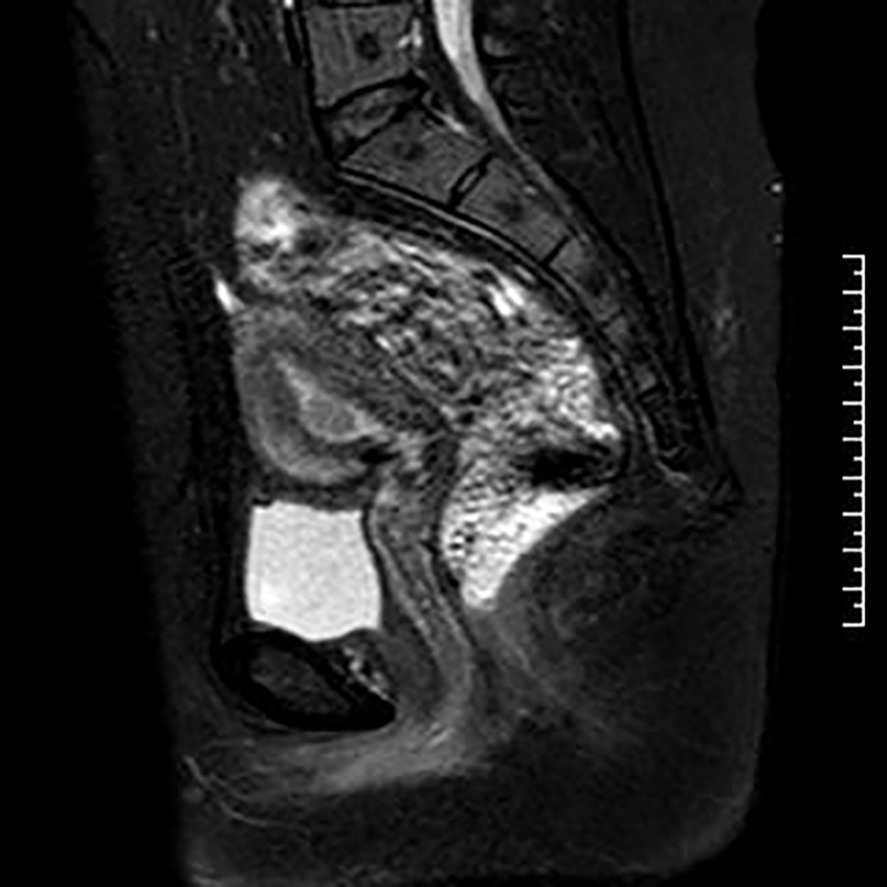
MRI at diagnosis.

The patient was a 31-year-old multiparous woman who underwent D&C in November 2021 due to abnormal uterine bleeding. The postoperative pathology revealed endometrial atypical hyperplasia with polypoid growth and suspicious focal cancer (**Figure 1**). And immunohistochemistry indicated ER positive (90%), PR positive (90%), p53 (wild type), Pax-2 negative (local). Following the D&C procedure, a MRI of the pelvic region was performed to fully assess the uterine cavity which showed that the lesion was confined to the uterine cavity without myometrial invasion or cervical involvement (**Figure 2**). Eventually she was diagnosed with a highly-differentiated (G1) endometrial adenocarcinoma (grade IA). Notably, the patient’s body mass index was 27.2 kg/m2 (weight 75 kg, height 166 cm), and she had an irregular menstrual cycle of 30-40 days. Her grandfather died of lung cancer and she denied other family history of tumors and family history of diabetes.

## 3 The therapeutic intervention, follow-up and outcomes

**Figure 3:**
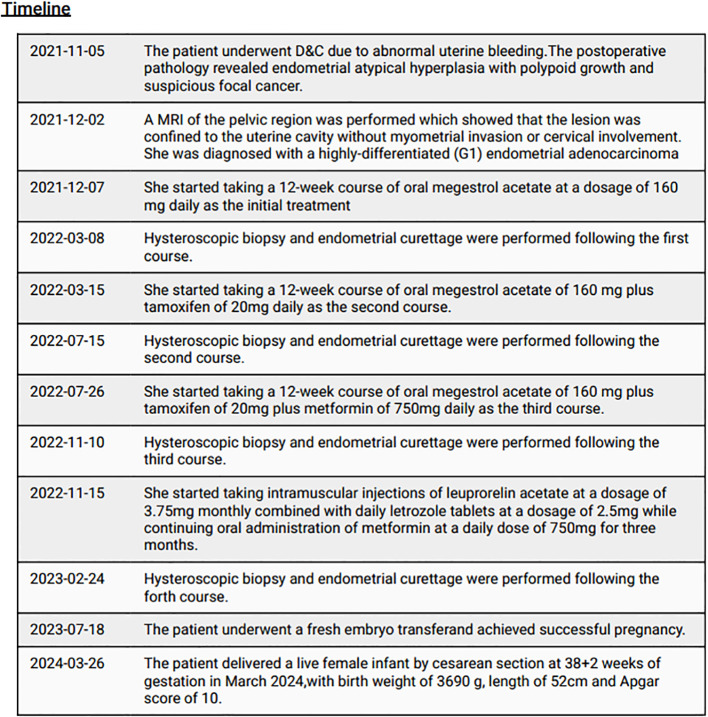
the timeline of diagnosis and treatment.

After counseling, the patient desired to preserve her fertility, thus high-dose progestin therapy was recommended. The timeline is shown in **Figure 3**. She underwent a 12-week course of oral megestrol acetate at a dosage of 160 mg daily as the initial treatment, followed by hysteroscopic biopsy and endometrial curettage. The postoperative pathology showed decidual-like changes in the endometrial stromal tissue with glandular atrophy observed in some areas (70%), consistent with medication-induced alterations. Immunohistochemistry analysis demonstrated positive estrogen receptor expression (60%), weakly positive progesterone receptor expression (5%), and localized Pax-2 positivity. In addition to the first course, Tamoxifen supplementation at a daily dose of 20mg was administered during the second course along with subsequent hysteroscopic biopsy and endometrial curettage procedures. The postoperative pathology showed decidual-like changes in the endometrial stromal tissue with glandular atrophy observed in some areas (90%). And immunohistochemistry indicated positive estrogen receptor expression (60%-70%) and individual cells showing progesterone receptor positivity. The third course involved an additional daily dose of 750mg metformin on top of the second course, resulting in extensive decidual-like changes in the endometrial stromal tissue along with glandular atrophy observed in approximately 85-90% of areas according to postoperative pathology findings. Immunohistochemistry analysis showed positive estrogen receptor expression (70%) and individual cells displaying progesterone receptor positivity. For the fourth course, medication was switched to monthly intramuscular injections of leuprorelin acetate at a dosage of 3.75mg combined with daily letrozole tablets at a dosage of 2.5mg while continuing oral administration of metformin at a daily dose of 750mg for three months. The follow-up pathology of hysteroscopic biopsy and endometrial curettage revealed that the majority of the glands in the endometrial tissue exhibited atrophy with secretory reaction and stromal decidual-like changes, demonstrating significant remission following treatment. Additionally, immunohistochemistry analysis indicated positive expression of ER and PR. The molecular test showed no mutation in the POLE gene and the molecular subtype was classified as non-specific molecular signature type (NMSP).

**Figure 4:**
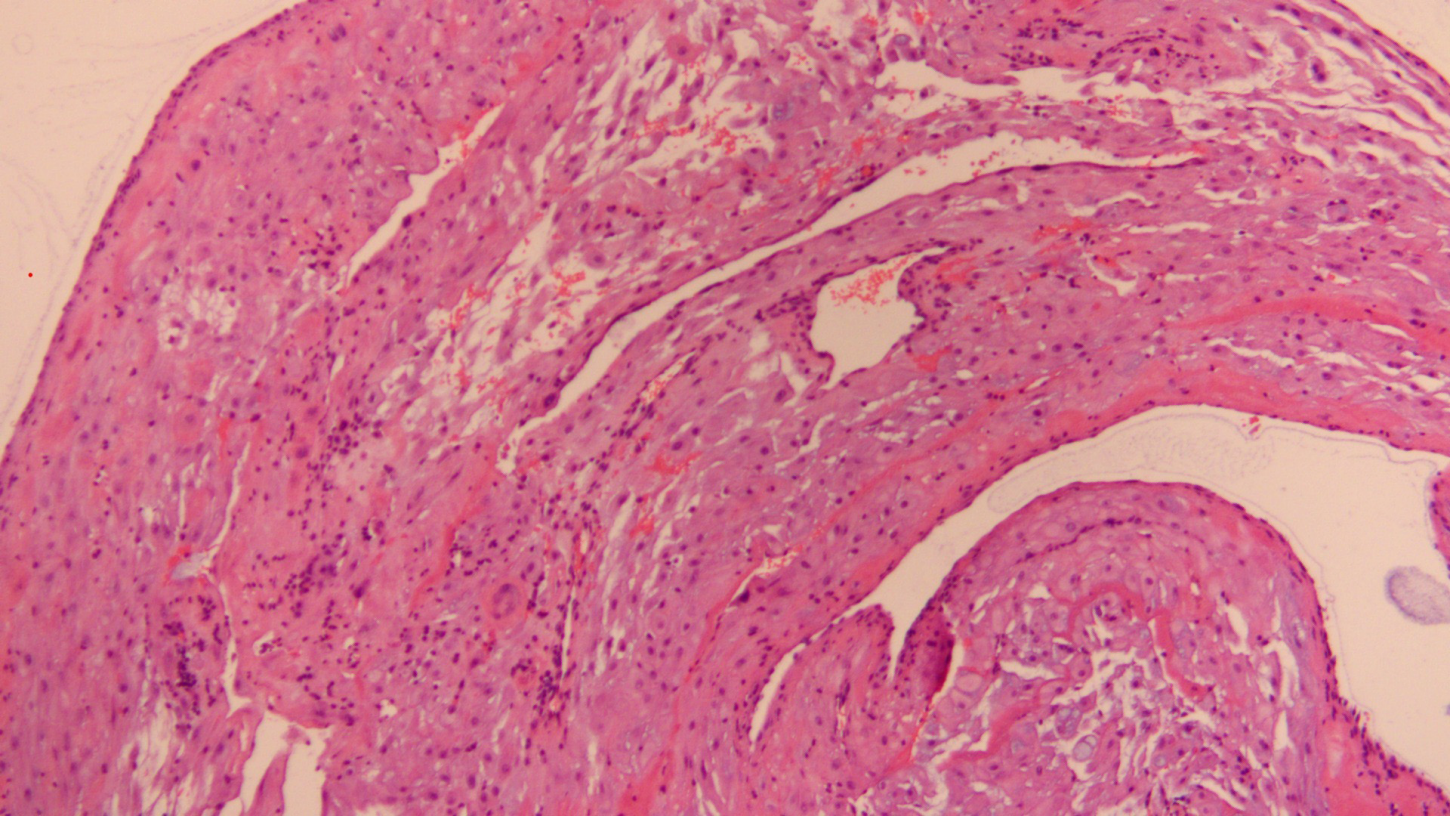
HE staining of the pathology section during cesarean section after treatment (10X).

In order to expedite conception, the patient chose to conceive by assisted reproductive technology. Her husband was 33 years old with a body mass index of 25.3 kg/m2 (weight 73 kg,height 170 cm). The husband’s semen analysis was normal.A mild stimulation protocol for ovulation induction was administered with letrozole at dose of 7.5mg daily from MC3 (artificial menstruation cycle Day3) and human menopausal gonadotrophin (HMG, Livzon, China) at dose of 225 IU daily from MC4 for 9 days in total. The follicular growth was monitored by transvaginal ultrasonography from the 5th day since gonadotropin administration by 2 days interval.When two or more follicles’ diameter reached 18 mm,human chorionic gonadotropin (hCG, Merck Serono, Germany) at dose of 6500 IU was administrated. The endometrial thickness was 7.5 mm at the time of hCG administration with following hormonal levels: LH 1.9 IU/L, P 0.8 ng/dL, E2 249 pg/mL. And 36 hours later, oocytes aspiration was performed under the guidance of transvaginal ultrasonography. Twelve oocytes were retrieved, and seven of them were fertilized and then cultured *in vitro*. A high-quality 10-cell embryo was transferred into the uterus 3 days after.The level of serum-hCG was 10887 mIU/mL 21 days after embryo transfer, and a single gestational sac was seen in the uterine cavity by transvaginal ultrasonography 35 days after embryo transfer. Routine prenatal examinations during pregnancy were normal. Considering the patient’s previous cesarean section delivery of a male infant in 2019, the pregnancy was terminated by cesarean section this time after multidisciplinary consultation and informed consent. Finally she delivered a live female infant by cesarean section at 38 + 2 weeks of gestation in March 2024,with birth weight of 3690 g, length of 52cm and Apgar score of 10. Meanwhile, curettage and endometrial biopsy was performed during the cesarean section.On one hand, the pelvic MRI in 2021 indicated that the endometrial cancer lesion was located near the uterine corner on the left wall of the uterus. On the other hand, the ultrasound images during current pregnancy suggested that placental placement occurred on the right wall of the uterus. Therefore, tissues from both uterine walls as well as both uterine corners were submitted for histopathology examination. The postoperative pathology report revealed minimal decidual tissue presence alongside abundant thick-walled vessels without significant atypical hyperplasia or malignant lesions (**Figure 4**).

## 4 Discussion

Endometrial cancer is one of the most common gynecological malignancies in developed countries (9), primarily observed during menopause. In recent years, there has been an increasing proportion of endometrial cancer cases diagnosed in individuals under the age of 40, accounting for approximately 2% to 14% of all reported cases (10). Previous successful conservative treatment cases for young endometrial cancer have predominantly involved nulliparous women. However, this particular case involves a young multiparous patient who underwent cesarean section delivery two years ago and subsequently received a diagnosis of endometrial cancer.

The uniqueness of this case lies in the absence of any identifiable risk factors for endometrial cancer, such as exposure to unopposed oestrogen, obesity, diabetes, advanced age, nulliparity, polycystic ovarian syndrome, and use of tamoxifen.A comprehensive umbrella review encompassing 171 meta-analyses from 1354 individual studies on 53 risk factors indicated that multiparity was associated with a reduced risk of endometrial cancer (11). However, the patient did not fit any of the above characteristics.Notably, she presented with endometrial polyps. Previous case series have reported varying malignancy risks in endometrial polyps (ranging from 0% to 15%), while one meta-analysis reported an overall prevalence of malignancy in endometrial polyps at 2.7% (12). Nevertheless, it remains unclear how long the patient had uterine polyps prior to diagnosis and whether her abnormal uterine bleeding was attributed to either the presence of polyps or underlying endometrial cancer.

The standards of each guideline are relatively unified on whether patients with early endometrial cancer can be treated with conservative treatment (1). In this case, the patient meets the following criteria for fertility-preservation treatment: 1. Young and strong fertility intention; 2. highly differentiated endometrioid adenocarcinoma; 3. imaging examination confirms localized lesion confined to the endometrium without invasion or metastasis; 4. positive expression of ER/PR; 5. molecular typing has no specific molecular characteristic (NMSP).

For patients with early endometrial cancer requiring fertility preservation and eligible for endocrine therapy, progesterone is the clinical first choice, and its therapeutic efficacy is highly related to the expression status of hormone receptors, especially the expression level of PR (13, 14). However, certain PR-positive patients are not sensitive to progesterone therapy (15). Although the pre-treatment PR positivity rate in this case was approximately 90%, it significantly declined to weakly positive (about 5%) after the initial course of treatment (oral administration of 12 weeks of megestrol acetate), indicating the development of progesterone resistance.

Tamoxifen, a selective estrogen receptor modulator (SERMs), is currently employed in the treatment of advanced and recurrent endometrial cancer. It exhibits tissue-specific actions by acting as an antagonist of estrogen in breast cancer tissue while displaying weak estrogenic characteristics in endometrial cancer tissue, thereby inducing PR expression (16). Additionally, a study conducted by Mao et al. (17) demonstrated that high-dose TAM also exerts regulatory effects on ERRα, which can inhibit the proliferation of endometrial cancer cells *in vitro*. Therefore, we incorporated tamoxifen into megestrol acetate in the second and third courses to enhance oral progesterone sensitivity while minimizing its side effects. However, despite these interventions, complete remission was not achieved based on pathological assessments and PR expression further decreased (with only individual cells showing positivity). Consequently, we contemplated discontinuing the progesterone treatment regimen.

The anti-tumor effects of gonadotropin-releasing hormone agonists (GnRH-a) are partially attributed to their ability to reduce estrogen levels in the body. Additionally, it binds to GnRH-R to effectively inhibit endometrial cancer cell proliferation (18, 19). Most of the endometrial cancer tissues express GnRH-R, suggesting that GnRH-aholds potential for treating PR-negative endometrial cancer. Current guidelines recommend combining GnRH-a with other drugs. Letrozole, an aromatase inhibitor, reduces estrogen production by inhibiting aromatase activity in ovarian and peripheral tissues; however, it is generally not administered as a standalone treatment option. Therefore, we discontinued the progesterone plus tamoxifen regimen in the fourth course and transitioned to a combination therapy involving GnRH-a and letrozole. Pathological examination following this course demonstrated significant remission.

It is worth mentioning that we have added metformin to the patients since the third course of treatment. Metformin, a conventional insulin sensitizer, was recently found to have both direct and indirect inhibition of EC and synergistic effects with progesterone (20–22). Patients with endometrial cancer often present with comorbidities such as polycystic ovary syndrome, overweight or obesity, and diabetes mellitus.Obesity and insulin resistance are independent adverse factors that not only compromise fertility preservation efficacy in endometrial cancer but also impact pregnancy outcomes (23). Therefore, proactive management of insulin resistance and body composition is imperative. Although the patient in this case did not meet the diagnostic criteria for obesity(BMI≥28.0kg/m2 according to Chinese standards), her BMI was 27.2kg/m2 —approaching the threshold for obesity diagnosis. Considering all these factors collectively, we introduced metformin into the therapeutic approach starting from the third course.

The recommended treatment duration for patients with endometrial cancer is 6-12 months, during which complete remission should be achieved (1). However, in our case, the patient did not achieve complete remission after 12 months of treatment but showed significant remission. Considering her strong desire to conceive, we decided to discontinue the treatment and assist her in achieving pregnancy as soon as possible after a multidisciplinary consultation. On the one hand, shortening the conception time can reduce estrogen stimulation and minimize the risk of endometrial cancer recurrence. On the other hand, pregnancy itself provides protection against endometrial cancer, which may be attributed to the female potential fertility or the biological process occurring early in pregnancy (24). Therefore, in order to improve the success rate, shorten the conception interval, and mitigate the higher risk of recurrence, we recommend the patients to conceive by assisted reproductive technology. Endometrial cancer is a hormone-dependent tumor, while the development of multiple follicles can significantly increase estrogen levels, potentially increasing the risk of recurrence. Therefore, we implemented a mild stimulation protocol using low doses of gonadotropin and letrozole to achieve an optimal balance between clinical pregnancy rates and recurrence rates.In early-stage endometrial cancer patients, long-term oral progesterone administration may exacerbate endometrial gland atrophy and stromal decidualization. Concurrently, hysteroscopy and curettage are typically required every 3-6 months during treatment. However repeated intrauterine procedures may cause damage to the functional layer of the endometrium, resulting in uterine adhesions and thin endometrium, etc. These factors are detrimental to successful implantation of fertilized eggs, potentially impacting subsequent pregnancies (25). The patient underwent a total of 36 weeks of oral progesterone treatment along with five hysteroscopic procedures and curettages within nearly two years from diagnosis until completion of treatment. Fortunately, she successfully conceived following one single embryo transfer.

Despite completion of childbirth, endometrial cancer patients remain at risk for recurrence and progression; therefore, surgical resection of the uterus is recommended (3). Upon the patient’s strong request for uterine preservation, a multidisciplinary consultation was conducted, leading to the decision of implementing close follow-up as a temporary measure.

## 5 Patient perspective

As a cancer survivor, it was not easy for my family or me to defeat it and have a second child. I was shocked when I was first diagnosed with cancer, because I had just had a baby two years ago. Fortunately, my doctors sincerely listened to my opinions, respected my choice of preserving the uterus, and showing the professionalism in every step of diagnosis and treatment. I am very grateful to doctors in various disciplines for their help in this process, which could not be achieved without them. Now I cherish my hard-won precious child and my own life.

## Data Availability

The original contributions presented in the study are included in the article/**Supplementary Material**. Further inquiries can be directed to the corresponding author.
