# Supplementary figures and images for: Case report: A successful live birth after in vitro fertilization and embryo transfer in a patient with endometrial cancer who was treated conservatively
Source: Front Oncol. 2024 Dec 11;14:1461216. doi: 10.3389/fonc.2024.1461216 (PMC11669042; doi:10.3389/fonc.2024.1461216)

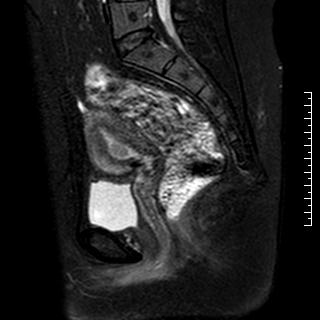

Supplement: Supplementary file 1 [file Image1.jpeg]
